# Gender, Obstacles, and Perspectives in the Career of Plastic Surgeons in Italy: a Survey-Based Analysis

**DOI:** 10.1007/s00266-026-05754-x

**Published:** 2026-03-17

**Authors:** Mariachiara Fabbri, Chiara Botti, Giovanni Botti

**Affiliations:** 1 Plastic Surgery Resident, Residency Program in Plastic Surgery, Università Cattolica del “Sacro Cuore”, Largo Agostino Gemelli 8, 00168 Rome, Italy; 2 Plastic Surgeon in Private Practice, Villa Bella Clinic – Via Europa 55, 25087 Salò, Italy

**Keywords:** Plastic surgery, Gender, Surgeons life, Women

## Abstract

**Background:**

The Italian plastic surgery workforce is undergoing a demographic shift, with a growing proportion of women entering the field. Despite this, little is known about gender-specific challenges such as work-life balance, discrimination, and career progression. This study aimed to compare the professional experiences of male and female Italian plastic surgeons.

**Methods:**

We conducted a cross-sectional online survey of Italian board-certified plastic surgeons and residents. The questionnaire addressed demographics, work-life balance, training opportunities, burnout, and professional challenges using multiple-choice, Likert-type items, and open-ended responses. Data were analyzed descriptively and stratified by gender and age.

**Results:**

A total of 430 surgeons responded (22.6% women, 77.4% men). Women were most represented in the 30–39 age group, while men predominated in the 40–49 group. Gender-based disparities emerged: 62.7% of women with children reported career-related delays in childbearing (vs. 32.7% of men), and 61.3% reported difficulties reconciling work and family life (vs. 44.1% of men). Nearly all respondents who felt excluded from career dynamics (96.7%) or experienced sexist comments (83.3%) were women. Universal stressors included burnout (64.7%), fiscal issues (59.4%), aggressive competition (63.8%), and medico-legal risks (82.5%). Young surgeons identified insufficient exposure to aesthetic surgery and limited first-operator cases as key training gaps.

**Conclusions:**

Although women are increasingly represented in Italian plastic surgery, they continue to face substantial barriers in work-life balance and career advancement. Shared systemic issues, burnout, fiscal pressures, and medico-legal risks, further burden all surgeons. Targeted action by professional bodies is essential to promote equity, improve training, and strengthen professional support.

**Level of Evidence IV:**

This journal requires that authors assign a level of evidence to each article. For a full description of these Evidence-Based Medicine ratings, please refer to the Table of Contents or the online Instructions to Authors www.springer.com/00266.

## Introduction

The number of plastic surgeons in Italy is experiencing a significant increase, with a notable rise in training positions from 21 in 1992 [1] to 114 in 2024 [2]. Concurrently, the number of female physicians enrolling in plastic and reconstructive surgery residencies has also grown, mirroring the general increase in female enrollment in medical faculties. While women constituted only 13.5% of plastic surgeons in 2014–2015 [3], this demographic shift raises important questions about the specific challenges and experiences faced by female surgeons in the field [4].

This study was prompted by a desire to investigate potential gender-related disparities in Italian plastic surgery. We aimed to understand the unique difficulties encountered by female surgeons compared to their male counterparts, including issues related to career-life balance, maternity, acceptance by colleagues and patients, and the influence of gender on their training pathway. Furthermore, we explored professional burnout within the community, examining its causes, its relationship with patients and fiscal issues, and drawing a comparison between hospital and private practice settings.

A review of existing literature revealed a scarcity of comprehensive data on these topics, specifically within the Italian context. While some studies have explored the quality of life of plastic surgery residents and specialists [5–9] and the challenges of motherhood for female surgeons [10], no single study has yet integrated and compared all of the variables mentioned above. The purpose of this article is therefore to shed light on these often-overlooked aspects of a plastic surgeon’s professional life, which significantly contribute to their emotional well-being and, consequently, to their professional performance.

## Materials and Methods

We conducted a cross-sectional online survey targeting Italian board-certified plastic surgeons and residents. The survey instrument was designed to be comprehensive, including structured questions on demographics, work-life balance, training and career opportunities, burnout, patient interactions, economic and medico-legal issues, social media, and professional priorities. Both multiple-choice and Likert-type items were utilized, complemented by selected open-ended prompts to gather qualitative data. Categorical variables were summarized as counts and percentages, and comparisons were made based on gender, primary professional activity (aesthetic or reconstructive), and age.

## Results

**Fig. 1:**
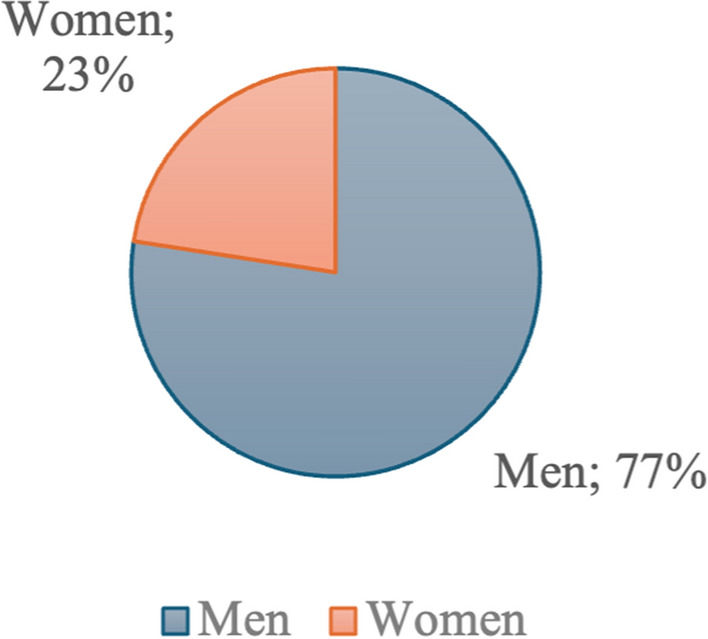
Participant demographics by gender

**Fig. 2:**
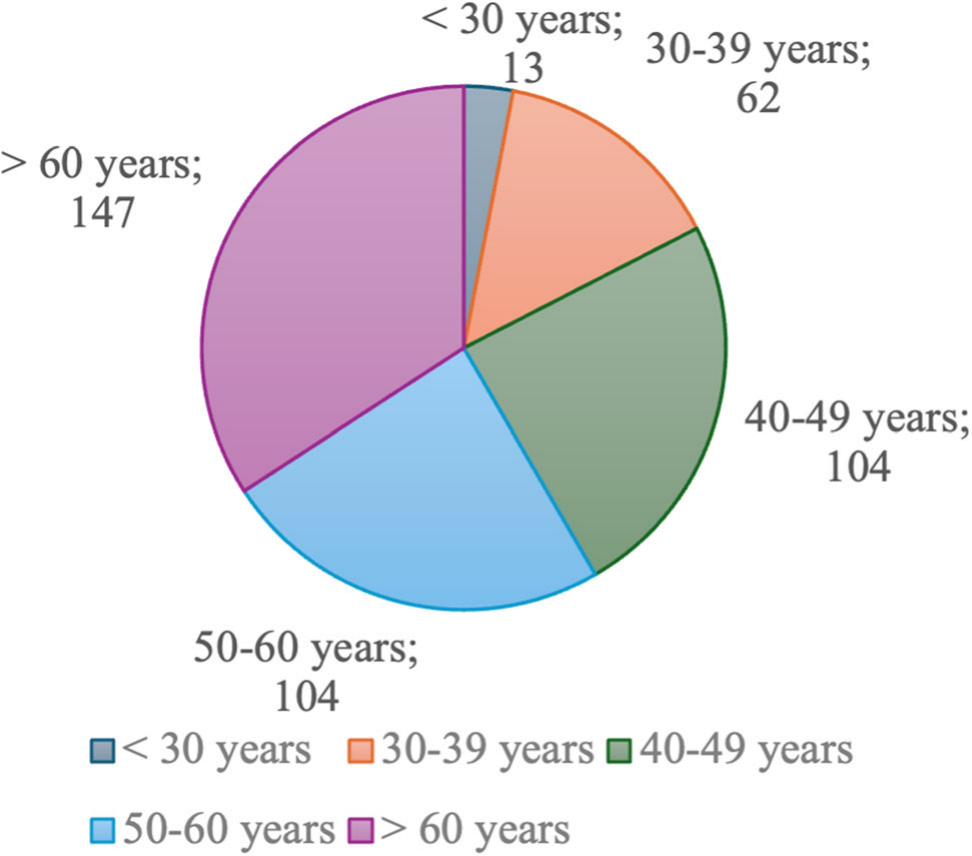
Distribution by age group

A total of 430 plastic surgeons responded to the questionnaire, comprising 97 (22.6%) women and 333 (77.4%) men. Regarding age, the majority of respondents were over 60 years old (34.2%), followed by those aged 50–60 (24.2%) and 40–49 (24.2%). Those aged 30–39 constituted 14.4% of the sample, while only 3% were under 30. However, female surgeons were most represented in the 30–39 age group, accounting for 39.2% of all female respondents. In contrast, male surgeons were most represented in the 40–49 age group, at 39.7% of all male respondents. The majority of participants worked in private practice (55.3%), while 11.2% worked in the public sector and 33.5% maintained a balance between both. Population characteristics are better described in Table 1 and 2 and Figs. 1 and 2.

**Table 1:** Participant demographics by gender

Gender	Number of respondents	Percentage (%)
Men	333	77.4
Women	97	22.6
Total	430	100

**Table 2:** Distribution by age group

Age group	Number of respondents	Percentage (%)
< 30 years	13	3.0
30-39 years	62	14.4
40-49 years	104	24.2
50-60 years	104	24.2
> 60 years	147	34.2

### Family Life and Professional Impact

**Fig. 3:**
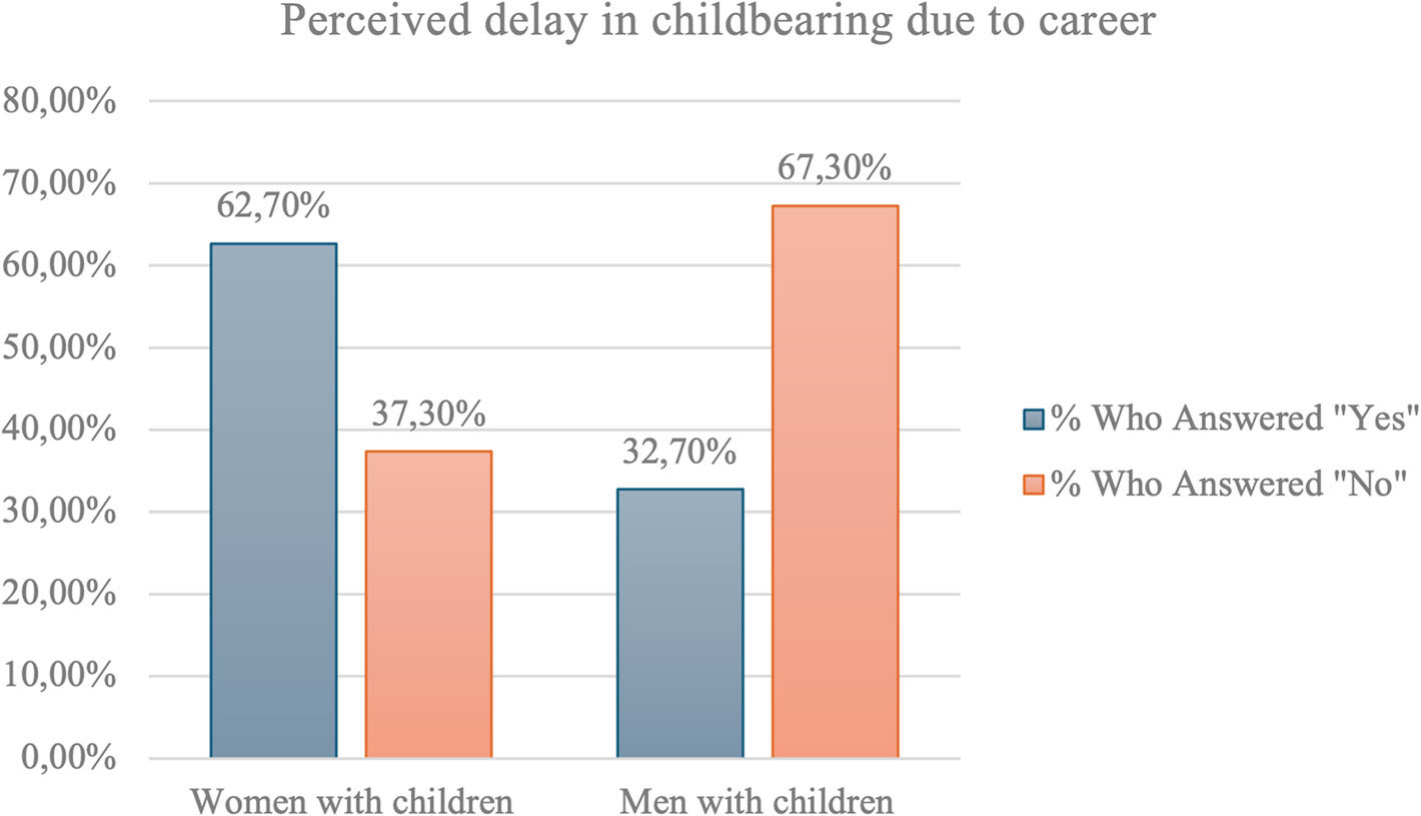
Perceived delay in childbearing due to career

**Fig. 4:**
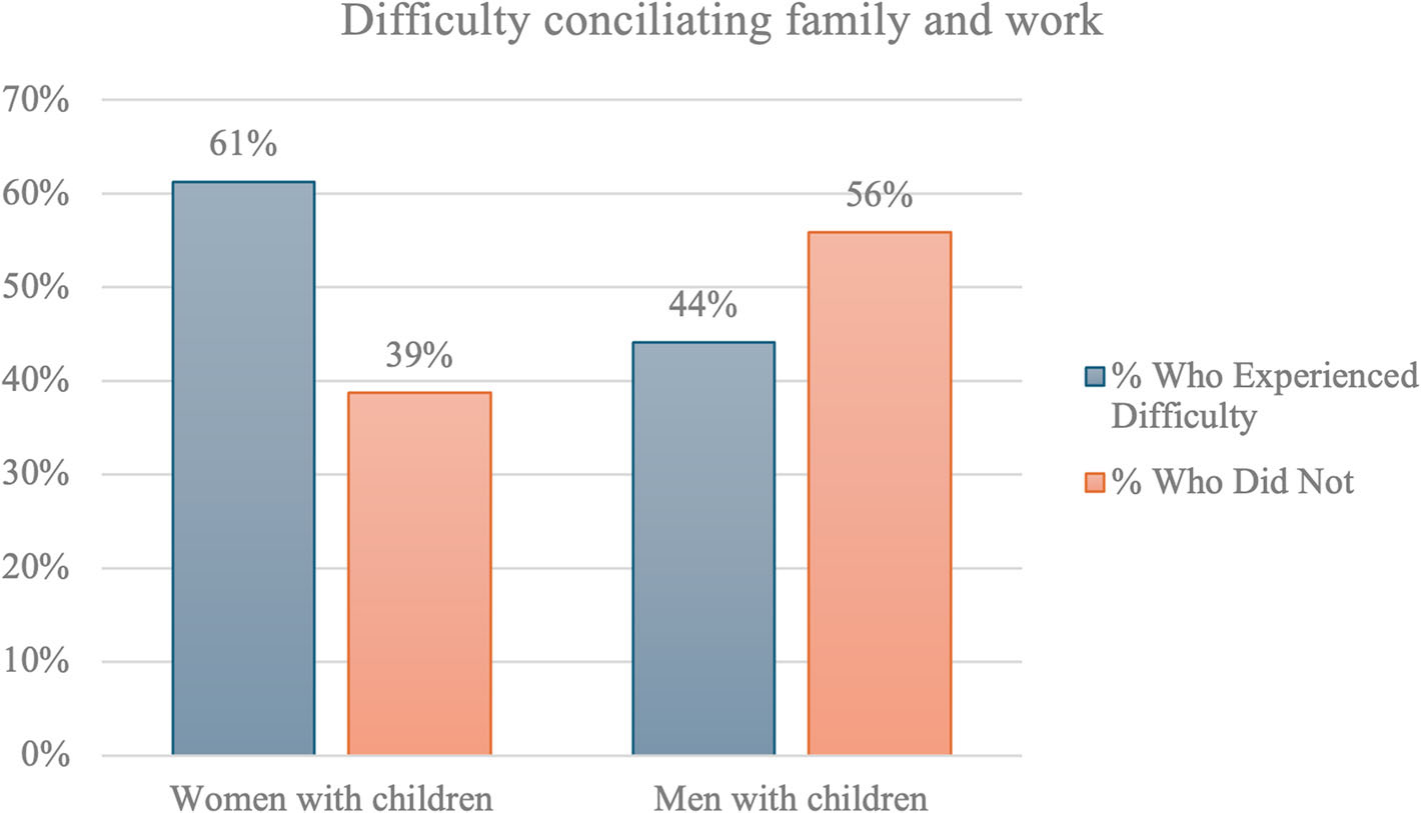
Difficulty conciliating family and work

- Impact on childbearing (Fig. 3): Of the respondents with children (72.3% of the total sample), 51.2% believed their professional path delayed the birth of their children. When stratified by gender, this perception was significantly higher among female surgeons; 62.7% (32 of 51 women with children) reported a delay in childbearing, whereas only 32.7% of men (18 of 55 men with children) responded affirmatively.
- Work-life conciliation (Fig. 4): A total of 77.7% of respondents reported experiencing difficulties in balancing family and work, with 43.4% reporting difficulties "often" and 34.3% "most of the time." A gender-based analysis revealed that 61.3% of female surgeons (38 of 62 women with children) found it difficult "often" or "sometimes" to reconcile family and work, compared to 44.1% of male surgeons (30 of 68 men with children).
- Career as a barrier to having children: Among those who did not have children, 43.1% considered it a personal choice, while 36.2% cited the fear of career penalization. Of this latter group, women constituted 40.5%. However, when analyzed as a percentage of the total male and female participants, a higher proportion of men (28.2%) than women (19.0%) expressed concerns that having children could negatively impact their professional growth.

### Workplace and Gender Dynamics

**Fig. 5:**
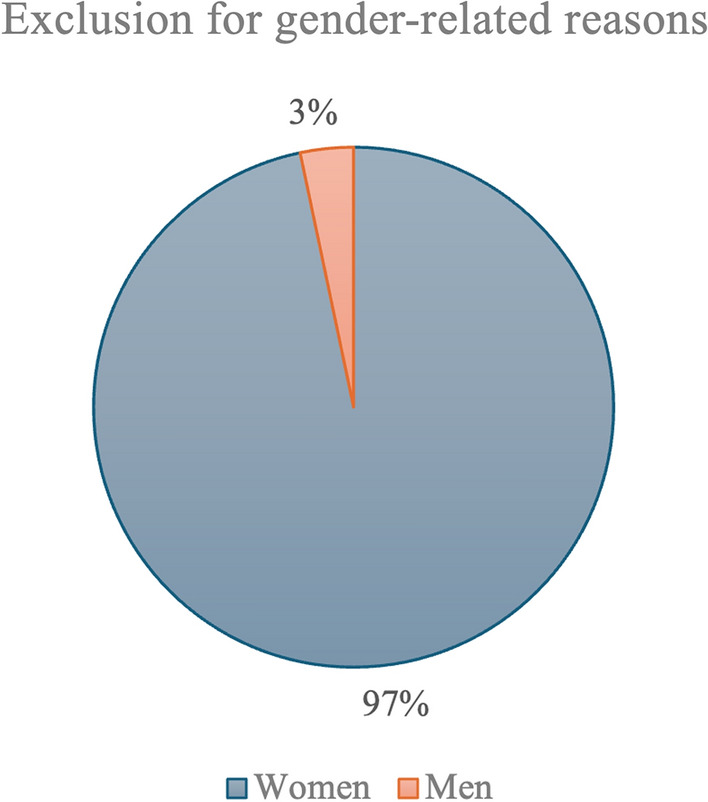
Exclusion for gender-related reasons

**Fig. 6:**
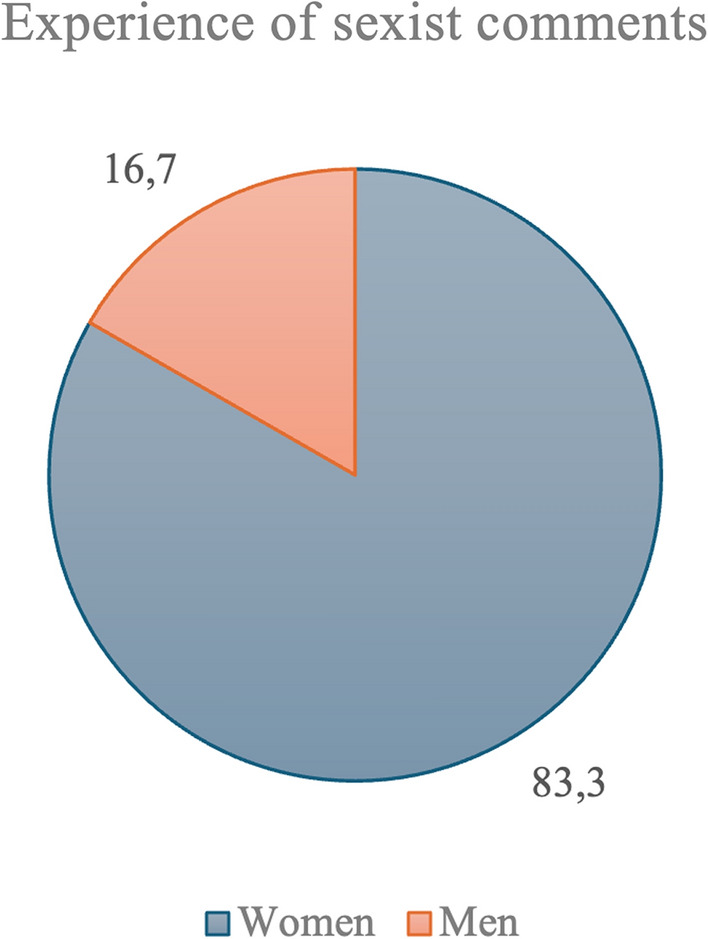
Experience of sexist comments

- Influence of gender on career (Fig. 5): While 76.5% of respondents believed their gender did not influence their career opportunities, a closer look at this group showed a slight male predominance (51.6% male vs. 48.4% female). Among the 22.8% who felt gender had influenced their training opportunities, the vast majority were women (37 of 38 respondents in this group). The most common open-ended responses cited limitations and disparities during residency, such as difficulty gaining access to key surgical procedures and a perception of being less valued for first-operator roles.
- Sexist comments (Fig. 6): While 75.8% of respondents reported never having received sexist or inappropriate comments, the data revealed a significant gender disparity in this regard. Among those who had received such comments (23.5% of the total), women constituted 83.3% while men constituted 16.7%. The women who reported receiving these comments were most prevalent in the 31–40 and 41–50 age groups.
- Exclusion from career dynamics: A total of 18.1% of respondents reported feeling excluded from career dynamics due to their gender. Of this group, 96.7% were women.

### Professional Burnout and Patient Interaction

**Fig. 7:**
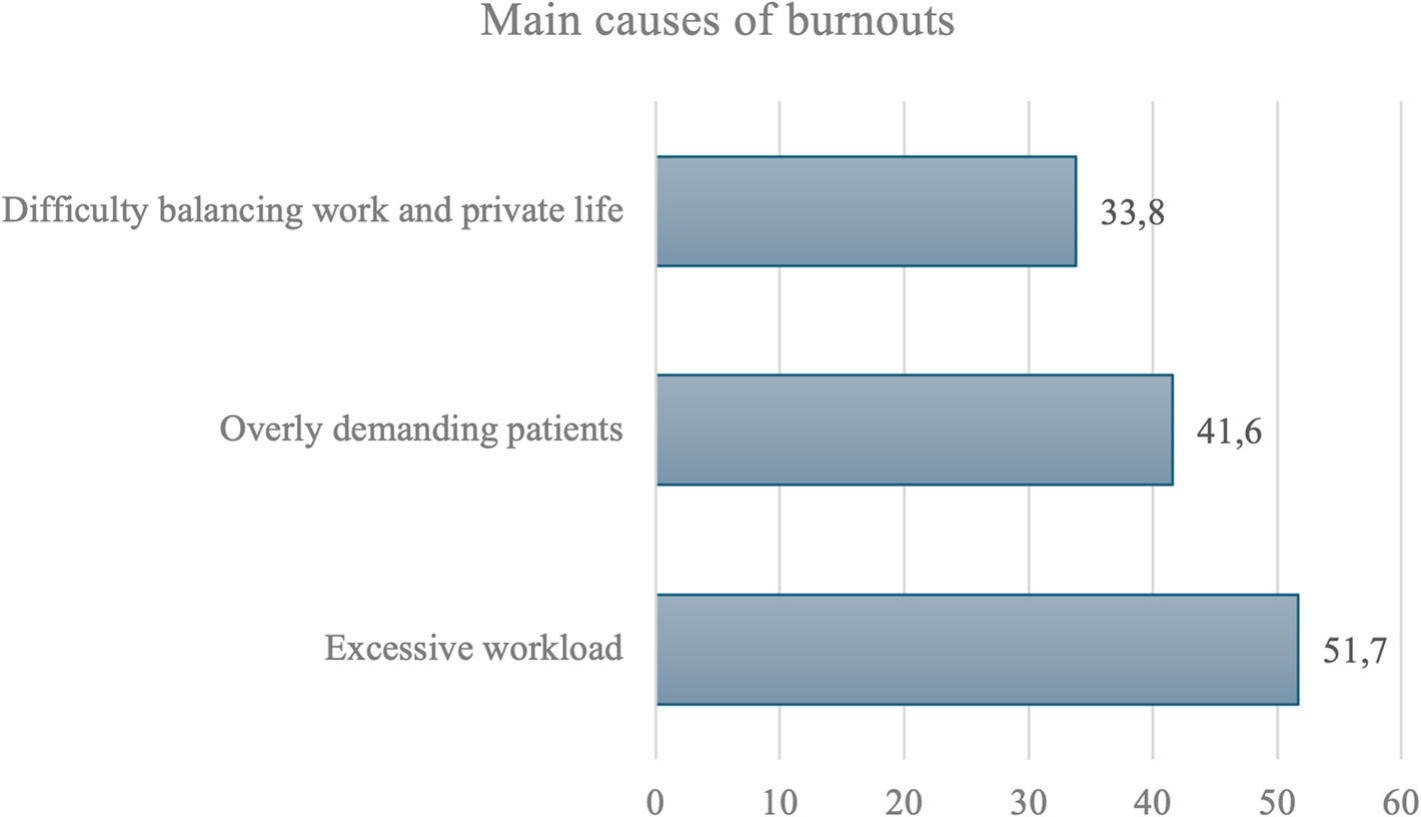
Main causes of burnouts

- Burnout factors (Fig. 7): A majority of respondents (64.7%) reported having experienced professional burnout. The most common contributing factors were excessive workload (51.7%), followed by demanding patients (41.6%) and difficulty balancing work and private life (33.8%).
- Support system: A significant portion of respondents felt only partially supported (45.3%) or not at all supported (28.4%) by colleagues in times of difficulty.
- Patient treatment: While only 10% of the total sample felt their gender influenced patient treatment, 73% of female surgeons specifically reported perceiving a different treatment from patients based on their gender.
- Unpleasant patient encounters: A total of 58.6% of respondents reported having experienced unpleasant episodes with patients. Based on open-ended responses, the most common causes included unrealistic patient expectations, manipulative behaviors (e.g., seeking free treatments or discounts), and managing patients with psychological issues like body dysmorphia.

### Economic and Professional Difficulties

**Fig. 8:**
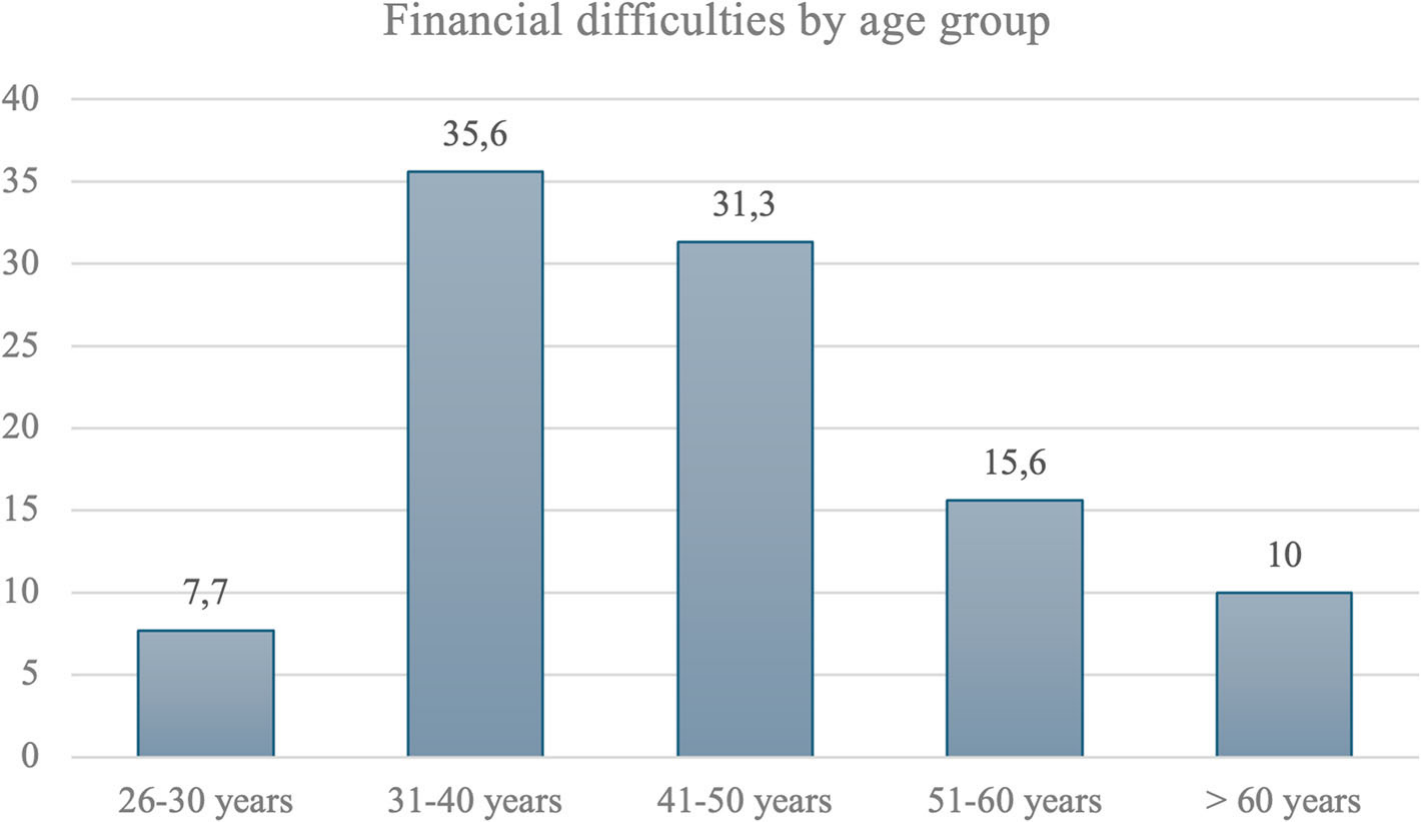
Financial difficulties by age group

**Fig. 9:**
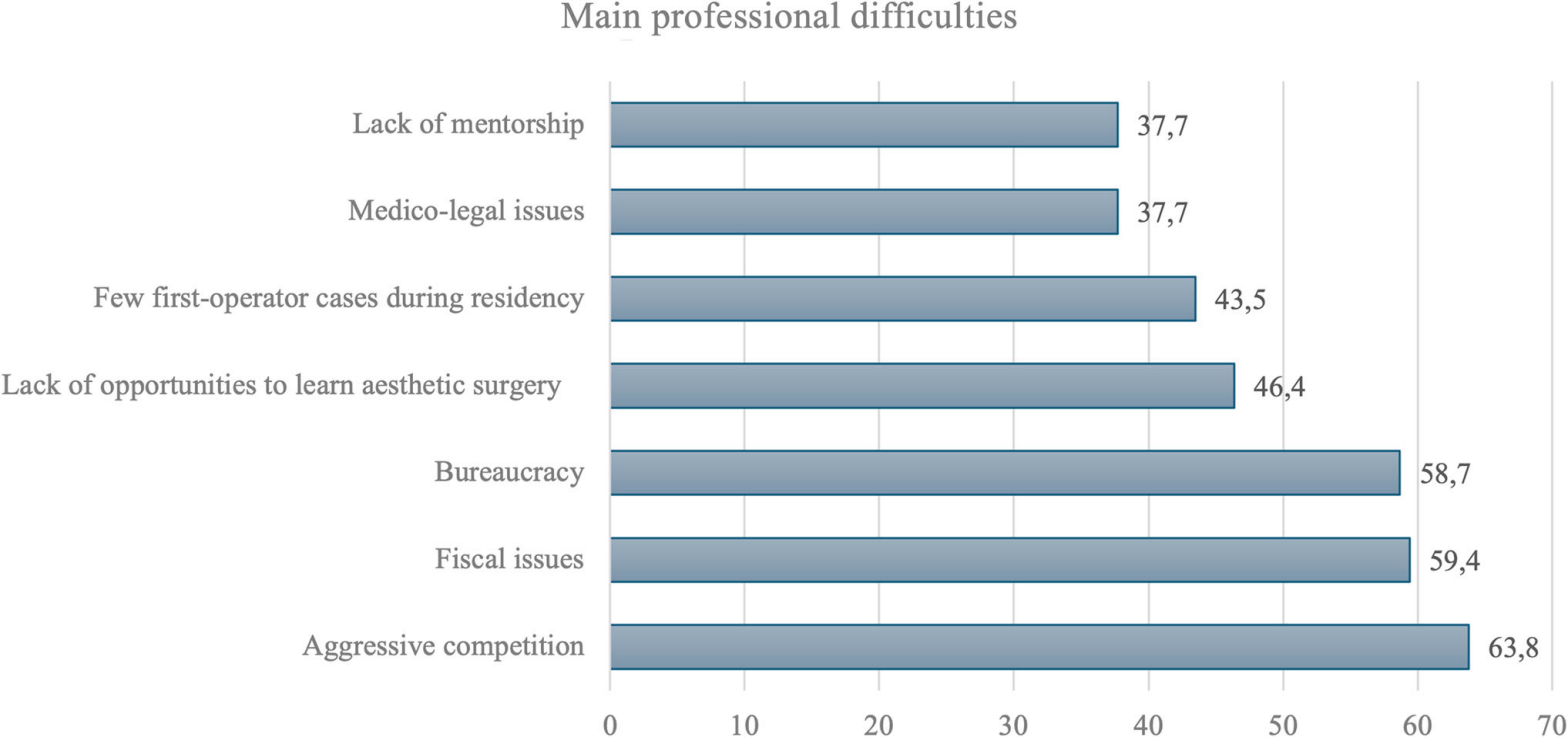
Main professional difficulties

**Fig. 10:**
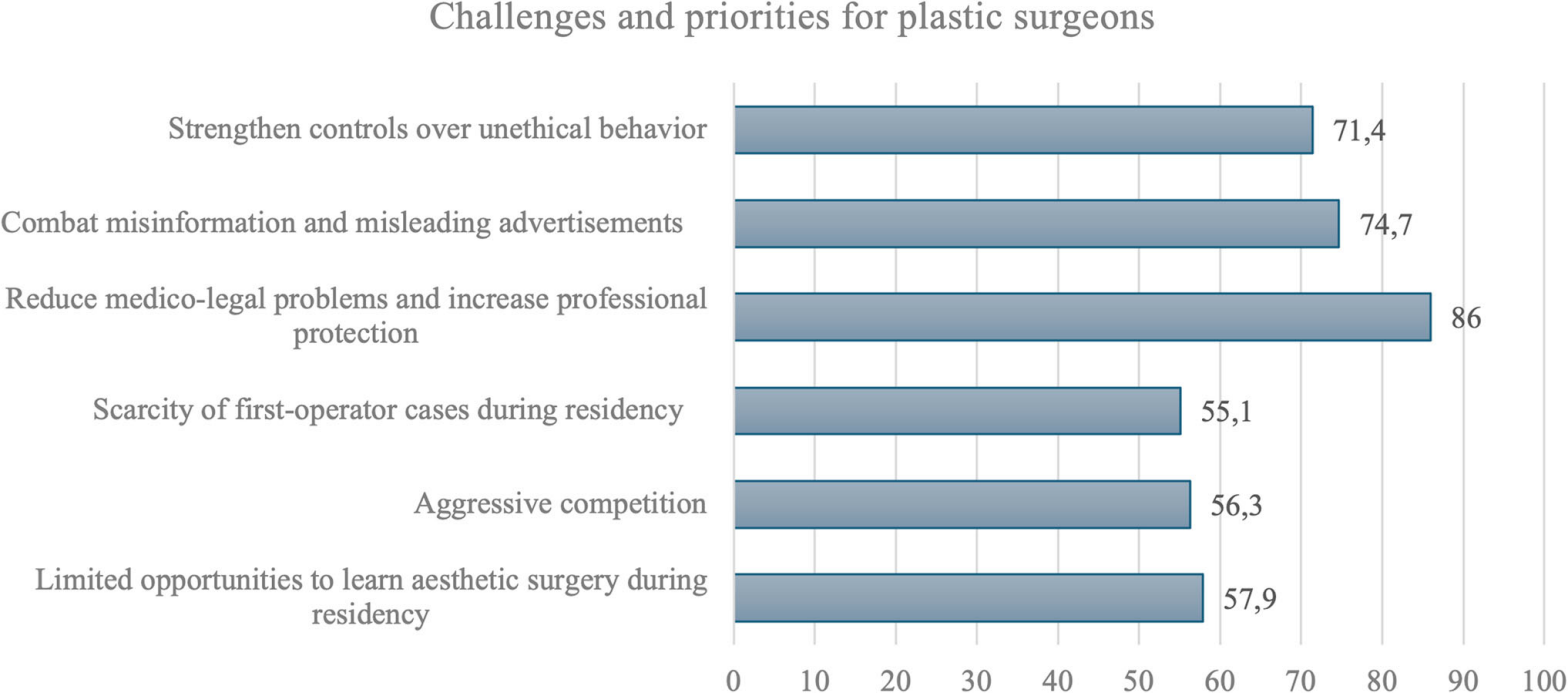
Challenges and priorities for plastic surgeons

- Financial management (Fig. 8): Among the 54% of respondents who reported difficulties in managing the financial aspects of their practice, the most affected age groups were 31–40 years (35.6%) and 41–50 years (31.1%). The breakdown by primary activity was 36.7% in aesthetic surgery, 28.9% in reconstructive surgery, and 34.4% in a mixed practice.
- Most common difficulties (Fig. 9): The most frequently reported difficulties for all surgeons were aggressive competition (63.8%), fiscal issues (59.4%), and bureaucracy (58.7%). Other significant challenges included limited opportunities to learn aesthetic surgery during residency (46.4%), few first-operator cases during residency (43.5%), a lack of mentorship (37.7%) and medical-legal issues (37.7%). Regarding this last one, 82.5% of respondents stated that medical-legal problems had a significant impact on their serenity and freedom of action (Fig. 10).

### Final Considerations

When asked about the major challenges for a young plastic surgeon today, respondents most frequently cited the limited opportunities for learning aesthetic surgery during residency (57.9%), aggressive competition (56.3%), and the scarcity of first-operator cases during residency (55.1%). To improve professional life, the top priorities identified were reducing medico-legal problems and increasing professional protection (86%), combating misinformation and misleading advertisements (74.7%), and strengthening controls over unethical behavior (71.4%).

## Discussion

This survey provides a unique and comprehensive insight into the current state of Italian plastic surgery, shedding light on persistent gender-related disparities and shared professional challenges. Consistent with prior studies on female surgeons [10, 11], our findings highlight significant obstacles related to work-life balance, career advancement, and professional discrimination. However, our study is unique in its direct comparison between male and female plastic surgeons, revealing a nuanced picture of the profession in Italy.

Moreover, according to the ISAPS Global Survey 2024 [12], Italy is estimated to have approximately 2188 plastic surgeons, representing about 3.7% of the global total. In this context, our sample of 430 respondents is particularly substantial, corresponding to nearly one-fifth of Italian plastic surgeons. Furthermore, given that nearly half of these professionals primarily engage in aesthetic surgery, and considering that the majority of our participants reported a focus on aesthetic practice, the survey likely represents a significant proportion of those dedicated predominantly to aesthetic procedures.

The data unequivocally indicates a lingering gender gap in the perception and experience of professional life. While both male and female surgeons reported encountering professional difficulties, women are disproportionately affected by issues related to work-family conciliation, career exclusion, and inappropriate behavior. For instance, a significantly higher percentage of women with children reported that their career delayed the birth of their children (62.7% vs. 32.7% of men) and found it difficult to balance family and work (61.3% vs. 44.1% of men). These figures suggest that while the field has become more accessible to women, the institutional and cultural frameworks may not have evolved sufficiently to fully support their dual professional and personal roles.

An intriguing paradox emerged from our data: a higher percentage of men (28.2%) than women (19%) expressed concern that having children could negatively impact their professional growth. This may suggest that while men perceive a potential career penalty for prioritizing family, women are already living this reality, as evidenced by the high percentage who reported actual delays in childbearing and difficulties with work-life balance. This underscores a systemic issue where the burden of family responsibilities still largely falls on female professionals, despite a growing awareness among their male colleagues of the potential professional consequences.

Furthermore, our analysis of qualitative and quantitative data revealed a stark gender disparity in discriminatory experiences. The fact that 96.7% of those who felt excluded from career dynamics were women, and that 83.3% of those who received inappropriate comments were women, is deeply concerning. It suggests that while the profession may not have overt barriers, subtle forms of bias and discrimination persist. This is further supported by the high percentage of female surgeons (73%) who perceived different treatment from patients based on their gender. These findings call for a concerted effort within the professional community to foster a more inclusive and equitable environment.

The survey also highlighted universal stressors affecting both genders, such as high rates of burnout (64.7%) driven by excessive workloads and demanding patients. The high percentage of respondents who cited aggressive competition, fiscal issues, and bureaucracy as major difficulties indicates that the profession itself, particularly in the private and mixed practice settings where most respondents work, is facing systemic pressures. The strong sentiment regarding the negative impact of medico-legal risks (82.5%) further points to a climate of professional anxiety that transcends gender lines. This concern warrants a direct discussion with government and institutional bodies to seek legislative and regulatory solutions that provide adequate professional protection, thereby mitigating a significant source of stress.

Our findings also align with the expressed desire for a more structured training pathway. The top concerns for young surgeons—limited opportunities to learn aesthetic surgery (57.9%) and few first-operator cases (55.1%)—indicate a gap between the training provided and the skills needed for a successful career in a highly competitive market. To address this, it would be a constructive and intelligent step for scientific societies to implement dedicated training programs and workshops focused on aesthetic surgery, building upon the nascent initiatives already in place. It is also imperative that residency programs are reformed to offer more opportunities for supervised first-operator cases, giving residents the chance to develop their surgical skills and confidence in a safe and structured environment.

The significant percentage (54%) of surgeons who reported financial difficulties also suggests a critical need for support. This finding indicates that professional education should extend beyond clinical skills to include business and financial management. We propose that scientific societies should develop specific educational modules or resources to guide young surgeons in managing their careers, including fiscal planning, private practice management, and economic strategy.

In light of these findings, we propose several improvements, echoing the priorities identified by the respondents themselves. The overwhelming consensus on reducing medico-legal problems and increasing professional protection (86%) is a clear call to action for professional associations and regulatory bodies. Additionally, our data supports the need to combat misinformation and unethical behavior (74.7%), which would not only protect patients but also improve the overall professional environment.

## Conclusions

This comprehensive survey of Italian plastic surgeons demonstrates that despite the growing presence of women in the field, significant gender-related disparities persist, particularly concerning work-life balance, career progression, and experiences with discrimination. Additionally, the profession as a whole faces systemic challenges such as burnout and medico-legal risks. Our findings underscore the need for targeted interventions by professional bodies to address gender-specific barriers and implement the priorities identified by the community, including enhancing legal protection, promoting ethical practices, and restructuring training programs to ensure equitable access to critical surgical experience. Ultimately, these measures are essential to foster a more equitable, supportive, and sustainable professional environment for all plastic surgeons in Italy.
